# People are different: tyrosine's modulating effect on cognitive control in healthy humans may depend on individual differences related to dopamine function

**DOI:** 10.3389/fpsyg.2014.01101

**Published:** 2014-10-06

**Authors:** Bryant J. Jongkees, Bernhard Hommel, Lorenza S. Colzato

**Affiliations:** Institute of Psychological Research and Leiden Institute for Brain and Cognition, Leiden UniversityLeiden, Netherlands

**Keywords:** tyrosine, cognition, dopamine, individual differences, genetics

## Introduction

The amino-acid tyrosine (TYR) is thought to modulate cognitive functions that are driven by dopamine (DA), as consumption of TYR enhances DA levels in the brain (Gibson and Wurtman, 1977; Cuche et al., 1985). It could therefore reverse decreases in DA level that are detrimental for cognitive performance (Muly et al., 1998; Goldman-Rakic et al., 2000; Nieoullon, 2002). So far, TYR has been considered not so much as an enhancer of healthy cognitive functioning but rather as a means to reduce the negative side-effects of dopamine-related pathologies, such as Parkinson's disease (Growdon et al., 1982; Lemoine et al., 1989), phenylketonuria (van Spronsen et al., 1996), depression (Gelenberg and Gibson, 1984), and attention deficit hyperactivity disorder (Wood et al., 1985). However, the outcomes were mixed: some patients reported significant improvements, while other did not. In clinical samples some variation in response may be explained by impaired processes such as DA synthesis, which would lessen or even completely prevent an effect of TYR. But even healthy samples differ in response to TYR supplementation, which suggests that clinically impaired DA function is not the only source of variation.

The focus of the present opinion article is not on clinical populations but on TYR effects on cognitive control in healthy humans. In healthy individuals, TYR has often been used to reduce the negative effects of conditions that deplete the brain's dopaminergic resources, such as extreme stress. The supply of TYR was found to reduce stress-induced impairments of working memory and attentional tasks, but more so in individuals who were particularly sensitive to the stressors (Deijen and Orlebeke, 1994; Shurtleff et al., 1994; Mahoney et al., 2007). Even without exposure to stress, administration of TYR has been shown to have an acute beneficial effect on task-performance thought to be related to DA, e.g., simultaneously performing multiple tasks (Thomas et al., 1999), the updating and monitoring of working memory (Colzato et al., 2013a), and inhibitory control (Colzato et al., 2014a). Taken together, in healthy humans TYR seems to work against what has been coined “ego-depletion”—the exhaustion of limited cognitive control (CC) resources (Baumeister et al., 1998). Demanding tasks may deplete the available control resources more, especially in individuals having fewer resources and/or those that suffer more from the situational demands, and TYR may be able to replete the missing resources to some degree. This possibility should not be surprising given that CC relies on DA (Cools, 2006). The hypotheses that DA is one of the depleted resources and TYR reverses its depletion are consistent with the idea that there is an optimal level of DA at which cognitive performance peaks while it suffers at lower levels (Muly et al., 1998; Goldman-Rakic et al., 2000; Nieoullon, 2002). Given that TYR raises the DA level, we argue that TYR can enhance cognitive performance in healthy individuals whenever one has a lower than optimal DA level.

Besides individual differences in the response to task-induced depletion, DA level also seems to vary between healthy individuals in a more stable and enduring fashion (Cools, 2006; Cools et al., 2008, 2009). This suggests that individuals differ in how far away they are from their optimum, i.e., some individuals have a lot of room for improvement, while others may already have an optimal, or even a higher-than-optimal DA level. We expect that individuals with an optimal baseline have little left of the enzyme called tyrosine-hydroxylase, which converts TYR into DA (Daubner et al., 2011). This means that they have little risk of overdosing from TYR supplementation, instead they should experience hardly any change in performance.

Given that individuals can vary in their response to TYR supplementation, it is necessary that future studies on TYR take into account individual differences, so to ensure that samples are comparable and results are generalizable. To this end we discuss a number of DA-related measures and factors that could predict or modulate the effect of TYR supplementation. This is by no means an exhaustive list; the aim of this opinion article is rather to point out and highlight some accessible predictors of DA function that may help to improve designing future TYR studies and making the analyses of their outcomes more informative. To this date, the individual differences discussed below have not yet been investigated in combination with TYR. However, based on literature that details their relation to DA function we argue that these individual differences will prove fruitful for future research.

## Indicators and modulators of DA function

At present, DA can only be measured (relatively) directly using positron emission tomography (PET), which is rather expensive and invasive as it involves injecting a radioactive substance into the bloodstream (Volkow et al., 2009). Noninvasive and cheap alternatives to estimate DA function exist, and some can be found in our eyes. The amacrine and interplexiform cells of the retina contain a high concentration of DA (Bodis-Wollner and Tzelepi, 1998; Witkovsky, 2004), and disorders associated with DA dysfunction have been related to abnormal color discrimination (Pieri et al., 2000; Tannock et al., 2006; Hulka et al., 2013). It has been proposed that deficits in color vision, particularly blue-yellow impairment, indicate a central hypodopaminergic state (Roy et al., 2003). This proposition is consistent with the recent finding that color discrimination predicts cognitive control, with better discrimination being associated with more efficient conflict-resolution in an auditory Simon task (Colzato et al., 2014b). Given the relation between color vision and DA level, we argue that color vision can predict the effect of TYR supplementation. Particularly individuals with impaired color vision could benefit from TYR, as they are likely to have less DA than non-impaired peers.

Another interesting aspect of our eyes is the spontaneous eye blink rate (EBR), which has been found to reliably indicate the striatal DA level (Karson, 1983). Specifically, a higher EBR is associated with more striatal DA. As expected, disorders related to abnormal DA function show atypical EBRs: Parkinson's disease is associated with decreased DA levels, and correspondingly with decreased EBRs (Deuschel and Goddemeier, 1998), while schizophrenia is associated with increased DA levels and increased EBRs (Freed, 1980). Also, EBR has been successfully used to predict individual differences in cognitive performance (e.g., Dreisbach et al., 2005; Colzato et al., 2007b, 2008a,b, 2009). Given the relation between DA and EBR, it follows that EBR can predict the benefit of TYR supplementation. We suggest that individuals with a low EBR, indicative of a low DA level, stand to benefit most from TYR, as it will bring their DA level closer to the optimum that is associated with peaking performance (Muly et al., 1998; Goldman-Rakic et al., 2000). Of further interest, striatal DA is thought to be particularly involved in cognitive flexibility (Cools, 2006)—the ability to update and switch between mental representations (Miyake et al., 2000). Therefore, EBR might predict improvement in performance especially on tasks that require a flexible mind.

Moving on to gene polymorphisms, the Val158Met-polymorphism in the catechol-O-methyltransferase (COMT) gene could also predict the effect of TYR supplementation, as this gene is involved in DA degradation in the prefrontal cortex. Specifically, the Met allele is associated with slower degradation of DA and high levels of prefrontal DA as the result, whereas the Val allele is associated with faster DA degradation and less prefrontal DA (Chen et al., 2004). As expected, several studies have shown Val-carriers to be less adept at cognitive tasks than Met-carriers (Egan et al., 2001; Mattay et al., 2003; Goldberg and Weinberger, 2004), which suggests that Val-carriers have lesser DA. This again implies that Val-carriers stand to benefit from TYR supplementation. Of further interest is the fact that prefrontal DA is tightly associated with cognitive stability (Cools, 2006), i.e., the ability to maintain task-relevant representations in the face of distractors or interference (Miyake et al., 2000). Given that the COMT gene modulates prefrontal DA level, its polymorphism could predict improvement especially on tasks that require cognitive stability.

Another candidate for modulating the effect of TYR supplementation is the C957T polymorphism in the DRD2 gene, which is linked to messenger RNA stability (Duan et al., 2003), leading to variation in extrastriatal D2 receptor availability (Hirvonen et al., 2009a) and striatal DA level (Hirvonen et al., 2009b). Specifically, T-carriers have reduced messenger RNA stability, which results in less striatal DA than homozygotic C-carriers. Correspondingly, T-carriers show less inhibitory control (Colzato et al., 2010, 2013b) and worse memory performance (Li et al., 2013). Also related to striatal DA is a second gene called DAT1, which is involved in DA reuptake (Lewis et al., 2001). Although there have been contradictory findings on how the varying number of base pair repeats in this gene relates to DA transporter (DAT) availability, recent studies suggest that 10-repeat homozygotes have lesser DAT availability, and consequently more striatal DA, than 9-repeat carriers (van de Giessen et al., 2009; Shumay et al., 2011). This is consistent with the finding that 10-repeat carriers are better than 9-repeat carriers at updating stimulus-response bindings (Colzato et al., 2013c), which is thought to be driven by DA (Colzato et al., 2007a,b). Given that both T-carriers of the DRD2 gene and 9-repeat carriers of the DAT1 gene are likely to have less striatal DA, we argue that especially they can benefit from TYR supplementation, again perhaps more so on tasks that require flexible cognition.

Interestingly, the aforementioned effect of the DRD2 polymorphism on inhibitory control and memory performance is stronger in older individuals than in younger ones (Colzato et al., 2013b; Li et al., 2013). This is consistent with the resource-modulation hypothesis (Lindenberger et al., 2008), which states that aging-related changes in neurophysiology enlarge the effect of polymorphisms on cognition. Given that DA systems deteriorate with old age (Volkow et al., 1998; Bäckman et al., 2000, 2006; Erixon-Lindroth et al., 2005), it is important to take characteristics such as age into consideration when investigating the modulating effect of TYR on DA and cognitive functions.

Last but not least is the tyrosine-hydroxylase (TH) gene, which codes for the enzyme that converts TYR into L-DOPA (Daubner et al., 2011). The C-824T-polymorphism in the TH gene has been shown to influence urinary excretion of norepinephrine, with carriers of the T allele excreting more norepinephrine (Rao et al., 2007). Horiguchi et al. (2014) found that in patients with schizophrenia the T allele is associated with increased TH transcription activity and higher IQ. These authors proposed that the resulting increase in norepinephrine levels in the brain may have protected the patients from cognitive decline. Higher levels of norepinephrine suggest higher levels of DA as well, since DA is the precursor of norepinephrine (Buu and Kuchel, 1979). Therefore, these findings have two interesting implications for studies on TYR. First, the higher TH transcription activity associated with the T allele in the C-824T polymorphism could mean that low baseline DA individuals who carry this allele can benefit especially from TYR supplementation, as more TYR can be converted into DA. Second, the idea that the T allele protects against cognitive decline in schizophrenia could imply that it does so in old age as well. In that case, the effect of this polymorphism on TYR supplementation might be strongest in older individuals, as might be the case with the DRD2 gene. This possibility again underscores the need to keep age and age-related changes in neurophysiology in mind when investigating TYR.

## Conclusion

The amino-acid TYR is a promising cognitive enhancer, yet studies on how TYR supplementation can benefit cognitive performance are still scarce. We suggest that future studies on TYR should take into account individual differences related to DA function, and to that end we have listed several indicators and modulators of DA that could predict the effect of TYR supplementation. It should be noted that these factors are unlikely to operate independently from each other. For example, individuals carrying the Val allele of the COMT polymorphism, which indicates a low prefrontal DA level, could benefit especially from TYR when also carrying the T allele of the TH gene, since that would allow more conversion of TYR into DA. As such, interactions between the presently suggested modulators should be taken into account, to achieve a better understanding of their implications for the effect of TYR supplementation.

### Conflict of interest statement

The authors declare that the research was conducted in the absence of any commercial or financial relationships that could be construed as a potential conflict of interest.
